# A CFD Study on Heat Transfer Performance of SiO_2_-TiO_2_ Nanofluids under Turbulent Flow

**DOI:** 10.3390/nano12030299

**Published:** 2022-01-18

**Authors:** Thong Le Ba, Gyula Gróf, Vincent Otieno Odhiambo, Somchai Wongwises, Imre Miklós Szilágyi

**Affiliations:** 1 Department of Inorganic and Analytical Chemistry, Faculty of Chemical Technology and Biotechnology, Budapest University of Technology and Economics, Muegyetem rakpart 3, 1111 Budapest, Hungary; vincent.odhiambo@mail.bme.hu; 2 Center for Energy Research, Konkoly-Thege Miklós út 29-33, 1121 Budapest, Hungary; grof.gyula@ek-cer.hu; 3 Department of Mechanical Engineering, Faculty of Engineering, King Mongkut’s University of Technology Thonburi, Bangmod, Bangkok 10140, Thailand; somchai.won@kmutt.ac.th; 4 National Science and Technology Development Agency (NSTDA), Pathum Thani 12120, Thailand

**Keywords:** nanofluids, ANSYS, CFD, turbulent

## Abstract

A CFD model was performed with commercial software through the adoption of the finite volume method and a SIMPLE algorithm. SiO_2_-P25 particles were added to water/ethylene glycol as a base fluid. The result is considered a new hybrid nanofluid (HN) for investigating heat transfer (HT). The volume concentrations were 0.5, 1.0, and 1.5%. The Reynolds number was in the range of 5000–17,000. The heat flux (HF) was 7955 W/m^2^, and the wall temperature was 340.15 K. The numerical experiments were performed strictly following the rules that one should follow in HT experiments. This is important because many studies related to nanofluid HT overlook these details. The empirical correlations that contain the friction factor perform better with higher Reynolds numbers than the correlations based only on Reynolds and Prandtl numbers. When temperature differences are moderate, researchers may consider using constant properties to lower computational costs, as they may give results that are similar to temperature-dependent ones. Compared with previous research, our simulation results are in agreement with the experiments in real time.

## 1. Introduction

Common liquids such as water, ethylene glycol, thermal oil, etc., are used in HT devices for different industrial applications. In the last few years, many studies have improved the HT to maintain more suitable HT in different thermal systems [1,2]. The hybrid nanofluid is a novel fluid with a hybrid nanofluid for high thermal conductivity (k) and stability. Much research has been conducted on HT through a tube with different nanofluids [3,4].

Hussein et al. investigated the impact of the cross area on HT and friction [5]. They found that, compared with circular and elliptical pipes, flat pipes have a notable increment of HT and pressure drop (PD). Additionally, they studied the augmentation of forced convection HT in an automotive cooling system [6].

In many studies, researchers have combined different nanomaterials to obtain novel composites with better properties [7,8] and nanofluids with higher HT efficiency [9,10,11,12,13,14]. Suresh et al. [15] synthesised Cu-Al_2_O_3_/water hybrid nanofluids with 0.1 and 2 vol% via a two-step method. Nanofluids’ k and viscosity (µ) increased with an increase in concentration growth. Madhesh et al. [16] studied the HT of Cu-Ti/water hybrid nanofluids with 0.1–1.0 vol%; they observed that the convective HT coefficient was maximum, with 48.4%, at 0.7 vol%.

Suresh et al. [17] presented a laminar convective HT under full development (FD) and PD through a heated flat pipe using Cu-Al_2_O_3_ hybrid nanofluids. They observed that the enhancement of the HT coefficient was 13.56% at *Re* = 1730, compared with water. Mosayebidorcheh et al. [18] studied the HT under a magnetic field and turbulent flow. The HT is proportional to the concentration and Reynolds number but inverse to the Hartmann number and turbulent parameter. Abbasi et al. [19] investigated the effect of the functionalisation technique on k and the stability of carbon nanotube/Al_2_O_3_ HNs. The functional groups significantly affected the k of HNs. The k enhancement was 20.68% for 0.1 vol%.

Labib et al. [20] chose the two-phase mixture model to the research convective HT of HN. Two kinds of base fluids and Al_2_O_3_ nanoparticles were used to determine the impact of base fluid on HT. The computational model was validated using carbon nanotubes with water nanofluids and compared with the data presented in previous research. It was found that ethylene glycol (EG) had better HT increments than water.

Sundar et al. [21] studied the HT of Fe_3_O_4_-MWCNT with a water HN under turbulent flow in a circular tube. Their experiment presented an HT increment of 31.10% with a cost of 1.18-times increase in pumping energy at 0.3 vol% and *Re* = 22,000 in comparison with water. The proposed correlations fit the experimental results. Using ultrasonication, Baby and Ramaprabhu [22] prepared Fe_3_O_4_/MWCNT and Fe_3_O_4_@SiO_2_/MWCNT nanofluids, which have k enhancements of 20% and 24.5%, respectively.

The turbulent flow in a circular duct and non-variable and variable properties of the working fluid have been studied. Generally, in the empirical formulas, the tube length-to-diameter ratio and the *Pr* number at mean flow temperature and *Pr_w_* number at wall temperature ratio account for the two phenomena. This study investigates the HT properties of SiO_2_-TiO_2_ HNs in water/EG under turbulent flow through a tube. Their properties are analysed using CFD simulation with commercial software. The TiO_2_ nanoparticles in the nanofluids have different sizes. The concentrations of nanofluid are 0.5–1.5 vol%. The water/EG mixture is the generally applied HT fluid to set the water/EG ratio according to temperature limitations, for example, in a four-season climate. Freezing can be avoided, and the pumping work is less, compared with a pure EG application.

## 2. Empirical Correlations and Fluid Properties

### 2.1. Theoretical Background

The HT research was conducted in at least two directions. The first was to perform experiments and then find and construct empirical and semi-empirical formulae (correlations) for solving engineering problems. The second was to analyse the HT phenomenon, investigating its physics, discovering the effects of working fluid properties, the wall–fluid interaction, and the validity of the known equations applied to describe the process. The results that yield the solutions for HT problems are based on a combination of experimental data and theoretical analysis. Earlier, physical experiments were the only way to determine HT subprocesses. Recent sophisticated CFD software offers reasonable alternatives, the so-called numerical experiments, for investigating HT problems related by determining the velocity and temperature fields in different arrangements. Independent of the type of experiment, the dimensionless numbers or groups resulting from the similarity theories (Buckingham pi theorem or differential similarity) prove crucial in HT calculations. Boussinesq assumptions are generally applied in solving the governing equations of the HT (mass, energy, momentum conservation). The matter properties variations are indirectly set up, usually by the proportion of the Prandtl numbers at two characteristic temperatures (average fluid and wall). Neither the differential similarity nor the pi theorem applies if the variation in properties is included in the fundamental equations.

The most investigated and basic arrangement is the horizontal tube with an isothermal wall or steady wall HF. One can also use these configurations in engineering formulae for HT calculation. It should be mentioned that the isothermal wall temperature, in most cases, can be maintained only in strict experimental conditions, and in practice, this assumption rarely exists. Nevertheless, the difference between the two basic approaches is nearly negligible for the Nu number in a turbulent flow. Promoting the numerical experiments (making CFD calculation), the boundary conditions can easily be established, and the results of the calculations are according to the desired conditions. That is one possible explanation for the observed variance, compared with the real experimental results.

### 2.2. Forced Flow in Circular Tubes

Forced flow occurs in multiple configurations, and one of the important engineering arrangements is the forced flow in the duct. The duct’s cross section may appear in various shapes, and in the engineering calculation, it is maintained through an equivalent circular diameter. Therefore, investigations generally deal with only the circular tubes.

Although the tests were carried out on a circular duct, it is advisable to recall that, in practice, the non-circular ducts cases can be handled well using the hydraulic (*D_eqH_*) and thermal (*D_eqT_*) equivalent diameter even in complex permanent cross-sectional structures. The definitions are as follows:

(1) $$D_{eqH}=4\;\frac{Area\;of\;duct}{Wetened\;perimeter\;}\;$$

(2) $$D_{eqT}=4\;\frac{Area\;of\;duct}{Working\;perimeter\;in\;heat\;transfer}$$

The hydraulic equivalent diameter should be used in the Reynolds number and the thermal in the Nusselt number.

**Figure 1:**
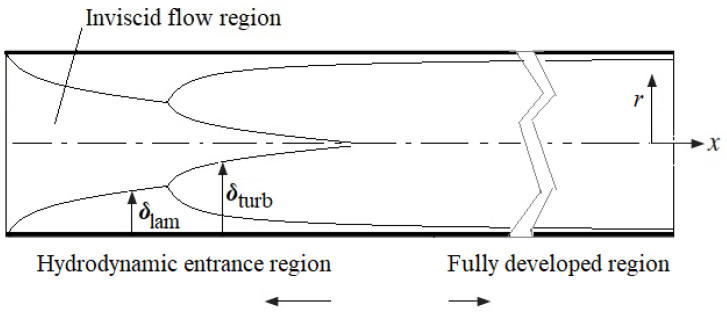
Hydraulically developing turbulent flow in the tube.

According to the *Re* number, the flows in a tube are characterised as laminar flow (*Re* < 2300), transitional flow (2300 < *Re* < 4000), and turbulent flow (4000 < *Re*). However, transitional flow is often considered part of turbulent flow in engineering practice. Moreover, depending on the distance from the entrance, there are different flow formations such as developing and (fully) developed flow regimes, as shown in Figure 1. Therefore, the local HT varies alongside the tube length, and some correlation formulae count this phenomenon. As both the velocity and the temperature vary from those of the entrance, there are hydraulically developing, thermally developing, and simultaneously developing flows.

#### 2.2.1. Turbulent Flow

The inner perturbation of fluid causes a turbulent flow. Different perturbations always exist in the fluid. When velocity is low, the viscous forces stabilise the flow pattern, but this stabilisation is not effective when the velocity increases and the local vortex formations result in a turbulent flow pattern. The laminar–turbulent transition never means a sharp borderline. There is always a particular transition zone. This phenomenon explains why the HT coefficient (Nu number) calculations perform less when the *Re* number is not much larger than the laminar *Re* number limit. There is always a particular length after the fully developed flow pattern is generated. Since this study subject is the turbulent forced flow, the *Re* number is greater than 5000 in all investigated cases.

In Handbook of Heat Transfer [23], there are 12 formulae for calculating the Nu number in the smooth tube for turbulent flow. The wide variation of the starting range for the *Re* numbers shows that generating a formula that accounts for the transition and the fully developed regime is not a simple task. The formulae can be characterised in two groups—either (a) the *ξ* friction factor is not included in the formulae or (b) it is, as shown in Table 1 and Table 2. Analysing all the formulae is beyond the scope of this study; therefore, we selected only three formulae to validate our numerical experiments for which the application range should fit 5000 ≤ *Re* and 3 ≤ *Pr*. The Dittus–Boelter (1) is generally used, while Sieder–Tate (6) accounts for the property variation, and Gnielinski (13) is based on the friction factor and also counts the tube length diameter ratio. The arrangements of the experiments should follow the limitations stated in the application ranges of the correlations considering flow development (hydraulic, thermal, and simultaneous). The other listed correlations might be useful for readers in further investigation or application of Nu number calculations.

According to the second law of thermodynamics, any methods or efforts made to intensify the HT result in an entropy generation, which manifests in the increased amount of work that must be performed to maintain flow configuration. The calculation of that increased work—practically the pumping work—is based on the determination of the friction factor of the flow arrangement, smoothness of the tube, and viscosity of the working fluid. When the working fluid property is subject to change, increasing the HT, this phenomenon—namely, the increased friction, always appears. The increased viscosity is the cost for the increased HT as the second law of thermodynamics states. The friction factor calculation might also be carried out according to several formulae. As the Prandtl–Kármán–Nikuradse friction factor definition is implicit, several value calculations are recommended and listed in Table 3 to overcome that difficulty.

### 2.3. Fluid Properties

SiO_2_-P25 nanoparticles and water/ethylene glycol mixture as base fluid were considered hybrid nanofluids (HNs), and the authors investigated these properties. The HNs in point were considered incompressible, Newtonian, and single-phase fluids. Table 4 illustrates the density (ρ), *k*, dynamic viscosity (*µ*), and specific heat (c*_p_*) of HNs obtained from the authors’ previous study [24].

**Figure 2:**
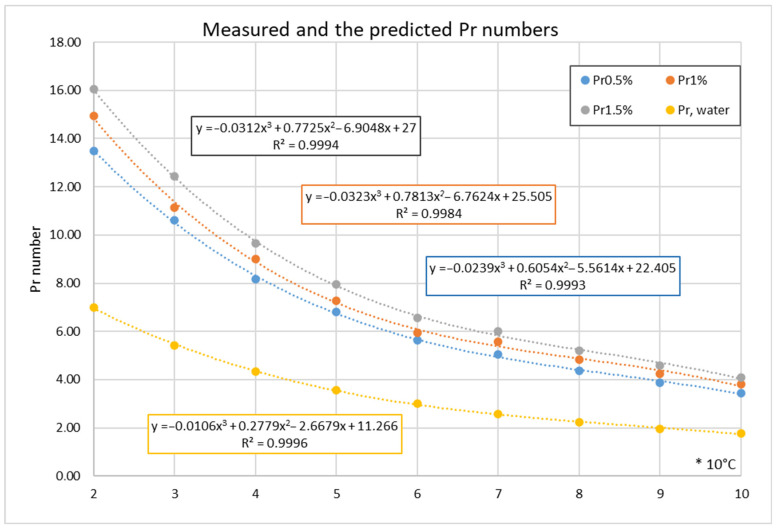
The measured and the predicted values of the *Pr* numbers for different concentrations.

The measurements were conducted at the temperature range from 20 °C to 60 °C. Some empirical formulae in Table 1 and Table 2 use the *Pr* numbers ratio as temperature correction. Therefore, the prediction was applied for the HN’s *Pr* numbers extension to 100 °C, as Table 5 reports. The prediction method can be summarised as follows: The ratio of the nano fluid’s *Pr* number against the water’s *Pr* number in the measurement range was calculated and extended by linear regression. Figure 2 visualises the measured and extended values. As shown, the extended *Pr* numbers are the natural continuity of the measured data as the third-order polynomial curve R^2^ values confirm. If needed, this prediction method works for other properties as well.

**Figure 3:**
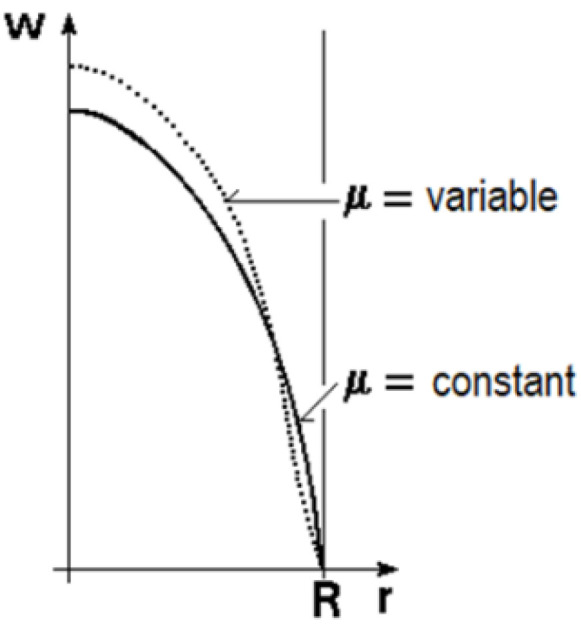
Velocity profiles for constant and variable viscosity [25], reprinted from Springer Nature open access article under the terms of the Creative Commons CC-BY license.

The empirical formulae should always follow the specific order related to the formulae. Generally, the characteristic length is specified, and the characteristic temperature is defined so that one should use it to determine fluid properties. When the formula has an extended application range, the *Re* and *Pr* numbers appear, and specific corrections may also count the specialties of the arrangement. Such correction could be the length-to-diameter ratio or the *Pr* numbers ratio. Figure 3 illustrates the effect that the properties variation may mean.

Since the mean fluid temperature plays a central role in the HT coefficient calculation, Equation (3) shows its calculation.

(3) $$T_{mean,f}=\frac{\int_{0}^{R}w\left(r\right)\cdot \rho \left(T\left(r\right)\right)\cdot C_{p}\left(T\left(r\right)\right)\cdot T\left(r\right)\cdot dr}{\int_{0}^{R}w\left(r\right)\cdot \rho \left(T(r\right))\cdot C_{p}\left(T(r\right))\cdot dr}$$

where *T_mean,f_* practically is the so-called completely stirred temperature.

## 3. CFD Analysis

### 3.1. Arrangements of the Numerical Experiment

The inlet temperature of the flow was 30 °C. The water was used for validating the CFD analysis. The inlet velocity was uniform. Static pressure conditions at the entrance and stagnation pressure conditions at the outlet were applied. The fluid’s velocity and temperature were varied so that the Reynolds number varied from 5000 to 17,000, and the flow was turbulent.

**Figure 4:**
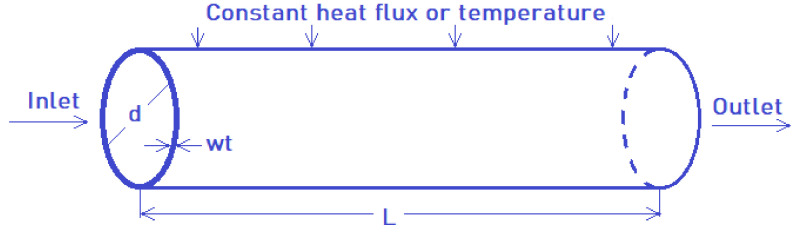
Scheme of the model.

The CFD study applied (a) constant HF of 7955 W/m^2^ and (b) a constant tube wall temperature of 67 °C. Figure 4 represents the arrangement, flow in a pipe with L = 1.5 m length, d = 16 mm hydraulic diameter, and wt = 2 mm wall thickness. We assumed that the flow in the tube is symmetrical and steady. The following equations were used for calculating the mass, energy, and momentum conservation with variable properties [26]:

(4) $$\frac{\partial \left({\bar{u}}_{i}\right)}{\partial x_{i}}=0$$

(5) $$\rho \left(\frac{\partial {\bar{u}}_{i}}{\partial x_{j}}+{\bar{u}}_{j}\frac{\partial {\bar{u}}_{i}}{\partial x_{j}}\right)=-\frac{\partial \bar{P}}{\partial x_{i}}+\frac{\partial }{\partial x_{j}}\left(\mu \frac{\partial {\bar{u}}_{i}}{\partial x_{j}}+\rho u_{i}^{\prime }{\bar{u^{\prime }}}_{j}\right)$$

(6) $$\frac{\partial \left(\rho \bar{T}\right)}{\partial x_{i}}+\frac{\partial \left({\rho \bar{u}}_{i}\bar{T}\right)}{\partial x_{i}}=\frac{\partial }{\partial x_{i}}\left(\frac{k}{c_{p}}\frac{\partial \left(\bar{T}\right)}{\partial x_{i}}\right)$$

**Figure 5:**
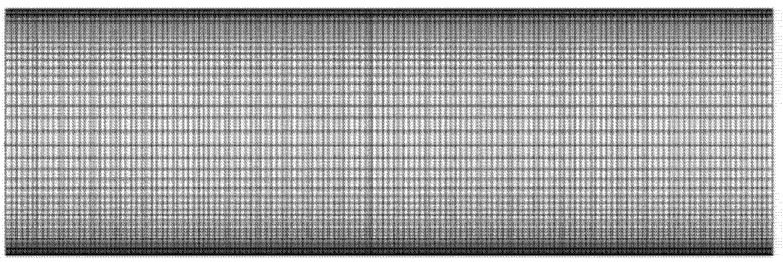
Mesh configuration.

ANSYS Workbench 16 (ANSYS Inc., Canonsburg, PA, USA) was used for this simulation research by solver strategy. The control volume approach provided the governing equations for the one-phase flow of the HNs. The geometry of the studied object was simulated as a circular duct (pipe), then the meshing was created based on the geometry, and boundary conditions were set up before the solving phase started. After the convergence reached the given criteria in the post-process phase, the HT and pressure drop were obtained at the computational domain. The applied mesh configuration is illustrated in Figure 5.

### 3.2. Grid Independence Test

**Figure 6:**
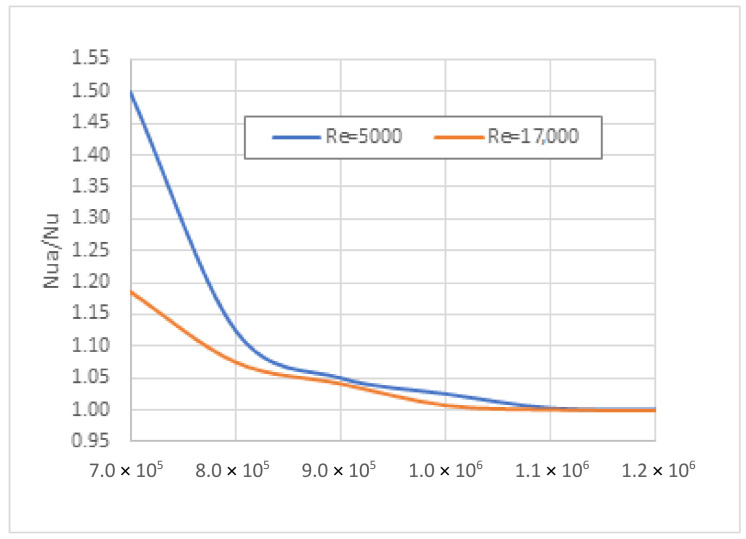
Nu number ratios against the mesh density.

The grid selection was tested using the result of the grid independence investigation. The investigated *Re* range was from 5000 to 17,000. The *Nu* numbers by the ANSYS calculation were determined for the *Re* = 5000 and *Re* = 17,000, with the mesh sizes of 7.0 × 10^5^, 8.0 × 10^5^, 9.0 × 10^5^, 1.0 × 10^6^, 1.1 × 10^6^, and 1.2 × 10^6^. Figure 6 shows the calculated ratio (Nu divided by Nu of the highest mesh) variation against the mesh density. We assumed that if mesh independence is proved at the end of the investigated range, the same mesh used is validated inside the *Re* range.

### 3.3. Fully Developed Flow

**Figure 7:**
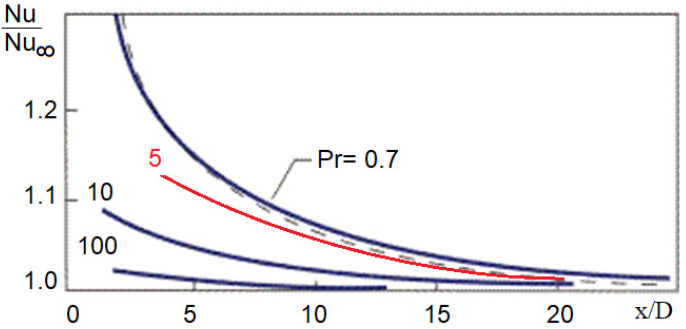
Effect of the *Pr* number on the development of HT in a tube.

In the formulae that account for the fully developed flow HT, the diameter-to-length ratio did not appear, except in (7) and (13), but (7) was not applicable due to its validity for low *Pr* numbers. The numerical experiment had to be performed for a long tube. The local HT variation was localised for a shorter length in turbulent flow configuration than the laminar flow; for calculating the entry length (*L_e_*), we used Equation (6) from [27]. However, in planning the experiment, the tube should be long enough to neglect that part. Figure 7 represents the local Nu number variation decrease from [23]. According to the investigated NFs, the *Pr* = 5 curve was assumed to determine that part of the length where the local Nu number varied. Practically, this means that our investigated zone started at L=~23·D=0.37 m from the tube entrance, as shown in Table 6, which lists the calculation results based on [27]. It is also worth mentioning that in [28], the proposed equation for hydrodynamic entry length is $Le=D\cdot 1.359\cdot Re^{0.25}$ and in [29], Le=~10·D. The smallest approximation for entry length is in [27,28,29] offers the largest one. We accepted this latter value.

(7) LeD=4.4·Re(16)

### 3.4. Velocity and Temperature Profiles

**Figure 8:**
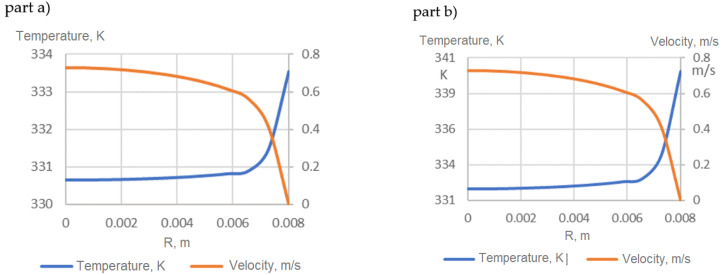
Velocity and temperature profiles in fully developed flow (**a**) constant HF (**b**) constant temperature of wall.

The velocity and temperature profiles of the HNs at the fully developed flow regime are represented in Figure 8 as an example for *Re* = 10,000. Part (a) shows wall subjects of constant HF, while part (b) shows wall subjects of constant temperature. According to sources in the literature, for example [23], in turbulent flow, there are no countable differences between part (a) and part (b) regarding the velocity profiles, but the temperature values are different; meanwhile, the shapes of the temperature profiles are also very similar.

### 3.5. Results

The following sections introduce our results based on the ANSYS calculation. The results are valid according to the limitations of the previous sections’ reports. Here, we mention again that the developing flow was not included in the results, compared with the correlations, because most are valid only for infinite length tubes.

#### 3.5.1. Constant HF with Non-Variable Properties, Developed Flow

**Figure 9:**
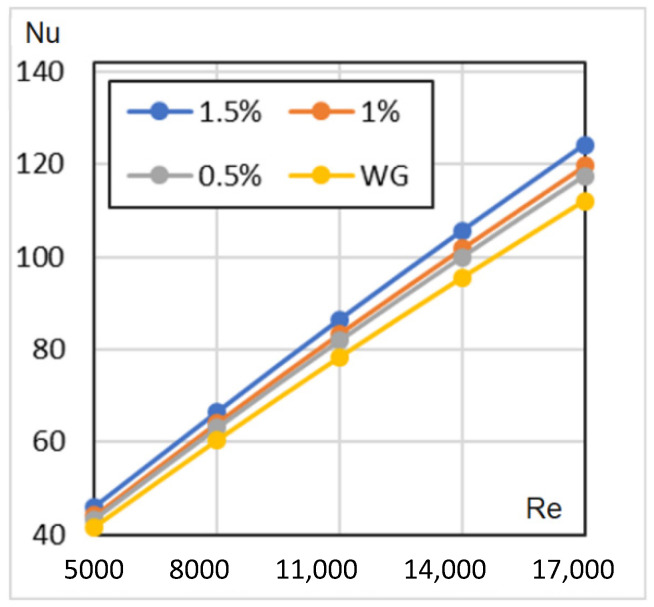
Nusselt numbers against Reynolds numbers for constant HF.

Figure 9 represents the numerical simulation results for a constant wall HF of Re = 5000…17,000. In agreement with sources in the literature, the 1.5% concentration HN has the highest Nu numbers.

#### 3.5.2. Constant Wall Temperature with Non-Variable Properties and Developed Flow

**Figure 10:**
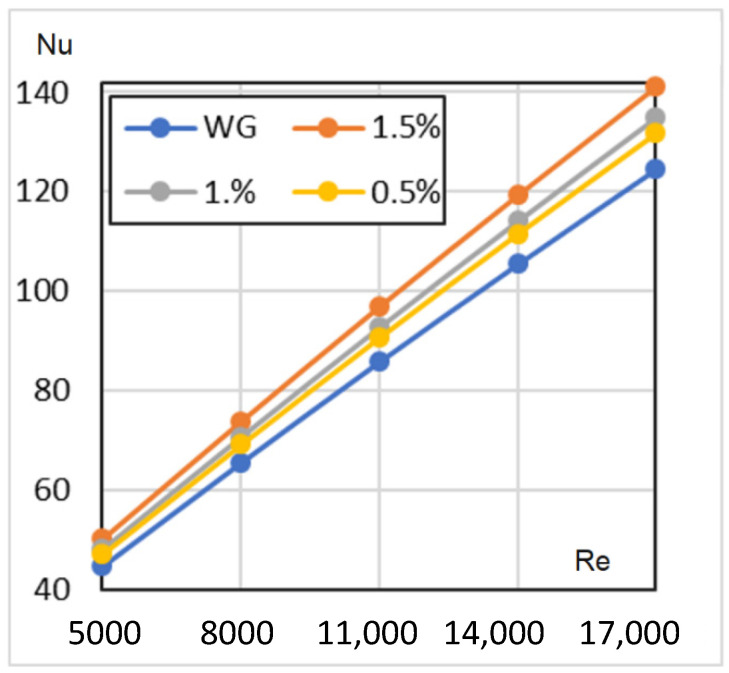
Nusselt numbers against Reynolds numbers for a constant wall temperature.

Figure 10 represents the numerical simulation results for a constant wall temperature of *Re* = 5000–17,000. In line with sources in the literature, the 1.5% concentration HN has the highest Nu numbers.

**Figure 11:**
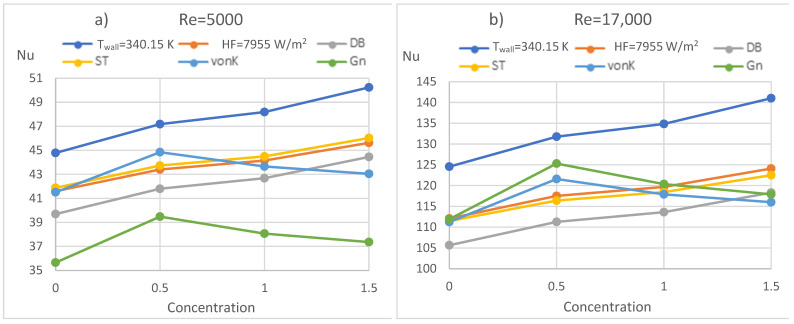
Comparison of the simulation results and correlations with (**a**) Re = 5000 (**b**) Re = 17,000.

Figure 11 introduces the comparison of the constant wall temperature and HF for (a) *Re* = 5000 and (b) *Re* = 17,000. Figure 11 shows the Nusselt numbers calculated by (1) Dittus–Boelter (DB), (6) Sieder–Tate (ST), (8) von Kármán (vonK), and (13) Gnielinski (Gn) correlations. The Temp curve represents Nu numbers for a constant wall temperature, and HF represents Nu numbers for a constant wall HF arrangement. The ST and DB correlations do not contain the friction factor, while the vonK and Gn include the friction factor. Since the tendencies are the same for all *Re* numbers in the investigated range, only the *Re* = 5000 and *Re* = 17,000 results are shown. The major observation can be summarised as follows:

- The Nu numbers of the constant wall temperature are 7–10% higher than the Nu numbers of the constant HF, and the difference increases as the *Re* number increases. The difference for constant HF and constant wall temperature is considered small in the literature, for example, in [23], but due to the greater temperature difference alongside the tube length, the HT is more intensive in the case of constant wall temperature. Moreover, the observed differences in the context of the accuracy of HT measurements are satisfactory;
- The DB correlation underestimates, while the ST correlation gives practically the same Nu numbers for constant HF;
- The ST correlation values are less than the numerical simulation result till *Re* = 14,000 and are above the simulation result only when *Re* = 17,000. It should also be mentioned that the ST correlation by [23] is valid only to *Re* = 10,000, but according to our results, it can be used at least to *Re* = 17,000;
- The correlation that contains the friction factor performs better at higher *Re* numbers. It should be mentioned that the application range for vonK starts from *Re* = 10,000, and for Gn, from *Re* = 2300, but vonK performs well below *Re* = 10,000 as well.

#### 3.5.3. Constant HF with Variable Properties

The properties of the fluids vary by temperature, but when the difference between the mean fluid temperature and the tube wall temperature is not high, the differences that the property variations may cause are nearly negligible. Table 7 illustrates the calculation results with CFD simulations using constant and variable properties. By considering the variable properties, the Nu number is generally higher by 1% in the investigated cases. 

#### 3.5.4. Constant Wall Temperature with Variable Properties

Table 8 illustrates the calculation results with constant and variable property CFD simulations. By considering the variable properties, the Nu number is generally higher by 2% in the investigated cases. The higher differences are due to the higher differences between the wall and mean fluid temperatures.

#### 3.5.5. HT in Flow Development

**Figure 12:**
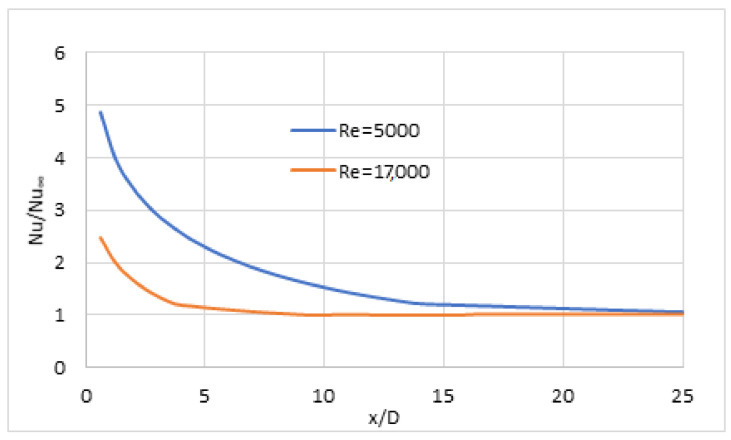
Local Nu number variation for 1.5 vol% NF.

Comparing the constant HF and constant wall temperature, one might find only limited differences according to the practical approach, as reported in [29]. Figure 12 reports the relative local Nusselt number variations (Nu/Nu∞) in constant HF for *Re* = 5000 and *Re* = 17,000 for 1.5 vol% concentrate NF. These results are in agreement with those shown in Figure 8 and Figure 9, based on [29]. The decrease in the Nu number is instant, and the development of the flow ends around *Le =* 10·*D* when the Reynolds number is above 10^4^, but for smaller Reynolds numbers, the flow development occurs at a somewhat greater distance. The authors advise the use of short tubes in the practical engineering application, considering flow development. A more intensive HT would occur than expected. Generally, this is not considered a problem and can be ignored.

#### 3.5.6. Friction Factors

**Figure 13:**
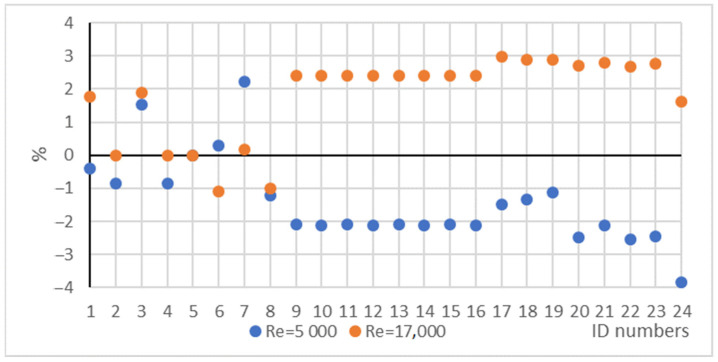
Percentage differences from the Prandtl–Kármán–Nikuradse friction factor.

As mentioned in Section 2.2.1, the friction factor has a significant role in determining the pumping power of the working fluids, and its value also appears in correlations for calculating the HT coefficient. Before easy access to the math software, several formulae were worked out to find the implicit Prandtl–Kármán–Nikuradse correlation. Table 9 shows all friction factor results we made using the formulae in Table 3, and the CFD calculation pressure drops in different cases. Figure 13 shows the percentage differences from the Prandtl–Kármán–Nikuradse friction factor calculation results for *Re* = 5000 and *Re* = 17,000. While the seven other correlation results are symmetrical, the CFD simulation results are “one sided”. When the *Re* value is 5000, the friction factors are less, and when the *Re* value is 17,000, the friction factors are higher than the references calculated by the Prandtl–Kármán–Nikuradse correlation. Our observation led us to not analyse these results in this study and leave it for future studies of the scientific community. The same results for No. 9–16 are due to the non-variable fluid properties.

**Figure 14:**
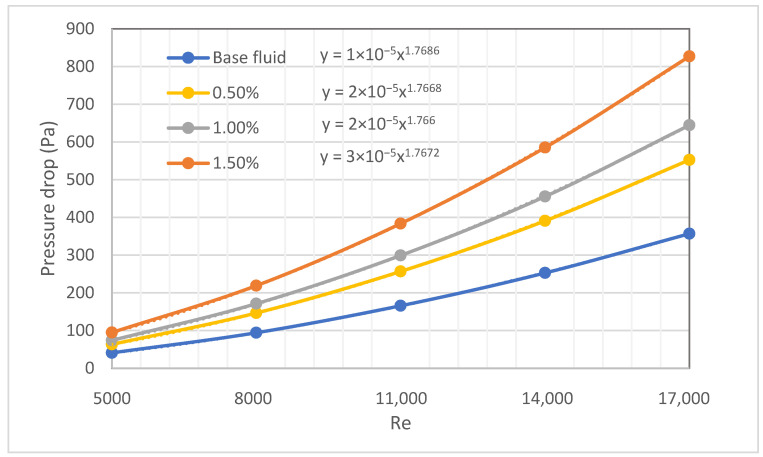
Pressure drops for SiO_2_-P25 hybrid nanofluids by CFD calculation.

The friction factor is more general than the pressure drop; Figure 14 shows the pressure drop of HNs obtained from simulation for non-variable properties. The results present how the pressure drop rises with higher *Re* numbers. If one substitutes the ξ(Re) function into the pressure drop calculation formula, the result is as follows:

(8) $$\Delta p=\frac{L}{d}\frac{1}{d^{2}}\frac{\rho }{2}{\left(\frac{\mu }{\rho }\right)}^{2}Re^{2}\xi \left(Re\right)$$

where the ξ(Re) is considered as $C\cdot Re^{m}$, according to our results, and the trendlines exponent 1.767, one obtains m = 1.767 − 2 = −0.233, which is close to the Blasius correlation exponent (−0.25), as shown in Table 3.

The pressure drop relatively increases by 56% and 137% at 0.5 and 1.5 vol%, respectively, because of the rise in viscosity.

## 4. Conclusions

In this research, the HT performance of SiO_2_-P25 nanofluids passing through a circular tube was investigated by CFD with constant HF and constant wall temperature. During the analysis, a comprehensive treatment of turbulent HT was offered by mentioning different known but often ignored details of the phenomenon. The following conclusions were drawn:

- Most of the simplifications in the context of the turbulent flow are acceptable, but one has to refer to the borders of the simplifications;
- When the real conditions do not meet the simplified circumstances, one should refer to the complex phenomenon;
- In the case of moderate temperature differences, the constant properties give similar results to the temperature-dependent case with lower computational cost;
- The Nusselt number and pressure drop increase with increasing concentrations of hybrid nanofluids and flow rate, in agreement with many experimental and theoretical studies.
- Compared with previous research, the simulated results are acceptable.

## Figures and Tables

**Table 1:** Correlations for Nu number based only on Re and Pr numbers [23].

Correlations (a)	Application Range
Dittus–Boelter (1) $Nu=\{\begin{array}{c} 0.023\;Re^{0.8}Pr^{0.4}\;for\;heating\\ 0.026\;Re^{0.8}Pr^{0.3}\;for\;cooling \end{array}$	0.7≤Pr≤120 $2500\leq Re\;\leq 1.24\cdot 10^{5}$ L/d>60
Colburn (2) $Nu=0.023\;Re^{0.8}Pr^{1/3}$	0.5≤Pr≤3 $10^{4}\leq Re\;\leq 10^{5}$
Drexel–McAdams (3) $Nu=0.021\;Re^{0.8}Pr^{0.4}$	Pr≤0.7 $10^{4}\leq Re\;\leq 5\cdot 10^{5}$
Gnielinski (4,5) $Nu=0.0214\;\left(Re^{0.8}-100\right)Pr^{0.4}$ $Nu=0.012\;\left(Re^{0.87}-280\right)Pr^{0.4}$	0.5≤Pr≤1.5 $10^{4}\leq Re\;\leq 5\cdot 10^{6}$ 1.5≤Pr≤500 $3\cdot 10^{3}\leq Re\;\leq 10^{6}$
Sieder–Tate (6) $Nu=0.027\;Re^{4/5}Pr^{1/3}{\left(\frac{\mu }{\mu_{w}}\right)}^{0.14}$	0.7≤Pr≤16 $700\leq Re\;\leq 10^{4}$
Hausen (7) $Nu=0.037\;\left(Re^{0.75}-180\right)Pr^{0.42}\left[1+{\left(\frac{x}{D}\right)}^{-\frac{2}{3}}\right]$	0.7≤Pr≤3 $10^{4}\leq Re\;\leq 10^{5}$

**Table 2:** Correlations for Nu number containing the friction factor [23].

Correlations (a)	Application Range
von Kármán (8) $Nu=\frac{\left(\xi /8\right)RePr}{1+5\sqrt{\xi /8}\left[\mathrm{Pr}-1+ln\left(\frac{5Pr+1}{6}\right)\right]}$	0.7 ≤ *Pr* ≤ 10 10^4^ ≤ *Re* ≤ 5 × 10^6^
Prandtl (9) $Nu=\frac{\left(\xi /8\right)RePr}{1+8.7\sqrt{\xi /8}\left(Pr-1\right)}$	0.5 ≤ *Pr* ≤ 5 10^4^ ≤ *Re* ≤ 5 × 10^6^
Friend–Metzner (10) $Nu=\frac{\left(\xi /8\right)RePr}{1.2+11.87\sqrt{\xi /8}\left(Pr-1\right)Pr^{-1/3}}$	50 ≤ *Pr* ≤ 600 5·10^4^ ≤ *Re* ≤ 5 × 10^6^
Pethukov–Kirillov–Popov (11) $Nu=\frac{\left(\xi /8\right)RePr}{C+12.7\sqrt{\xi /8}\left(Pr^{2/3}-1\right)}$ $C=1.07+\frac{900}{Re}-\frac{0.63}{1+10Pr}$	0.5 ≤ *Pr* ≤ 10^6^ 4000 ≤ *Re* ≤ 5 × 10^6^
Webb (12) $Nu=\frac{\left(\xi /8\right)RePr}{1.07+9\sqrt{\xi /8}\left(Pr-1\right)Pr^{1/4}}$	0.5 ≤ *Pr* ≤ 100 10^4^ ≤ *Re* ≤ 5 × 10^6^
Gnielinski (13) $Nu=\frac{\left(\xi /8\right)\left(Re-1000\right)Pr}{1+12.7\sqrt{\xi /8}\left(Pr^{2/3}-1\right)}\left[1+{\left(\frac{D}{L}\right)}^{2/3}\right]\phi_{T}$	0.5 ≤ *Pr* ≤ 2000 2300 ≤ *Re* ≤ 5 × 10^6^ $\xi ={\left(1.82log_{10}Re-1.64\right)}^{-2}$ $\phi_{T}={\left(\frac{Pr\left(Tmean\right)}{Pr\left(Twall\right)}\right)}^{0.14}$ For the case of constant fluid properties: D/L = 0, and Φ_T_ = 1.
Sandall et al. (14) $\frac{\left(\xi /8\right)RePr}{12.48Pr^{2/3}-7.853Pr^{\frac{1}{3}}+3.613\ln\left(Pr\right)+5.8+C}$ $C=2.78ln\left(\frac{Re\sqrt{\xi /8}}{45}\right)$	0.5 ≤ *Pr* ≤ 2000 10^4^ ≤ *Re* ≤ 5 × 10^6^

**Table 3:** Correlations for friction factor, [23].

Correlations	Application Range
Blasius $(1)\;\xi /4=0.0791Re^{-0.25}$	4 × 10^3^ ≤ *Re* ≤ 10^5^
Drew et al. $(2)\;\xi /4=0.00128+0.1143Re^{-0.311}$ $(3)\;\xi /4=0.0014+0.125Re^{-0.32}$	4 × 10^3^ ≤ *Re* ≤ 5 × 10^6^ 4 × 10^3^ ≤ *Re* ≤ 10^7^
Bhatti and Shah $(4)\;\xi /4=0.00128+0.1143Re^{-0.311}$	4 × 10^3^ ≤ *Re* ≤ 10^7^
Prandtl–Kármán–Nikuradse $(5)\;\frac{2}{\sqrt{\xi }}=1.7272\ln\left(\frac{Re\sqrt{\xi }}{2}\right)-0.3946$	4 × 10^3^ ≤ *Re* ≤ 10^7^
Colebrook $(6)\;\frac{2}{\sqrt{\xi }}=1.5635\ln\left(\frac{Re}{7}\right)$	4 × 10^3^ ≤ *Re* ≤ 10^7^
Filonenko $(7)\;\frac{2}{\sqrt{\xi }}=1.58\ln Re-3.28\;$	10^4^ ≤ *Re* ≤ 10^7^
Techo et al. $(8)\;\frac{2}{\sqrt{\xi }}=1.7372\ln\left(\frac{Re}{1.964\ln Re-3.8215}\right)$	10^4^ ≤ *Re* ≤ 10^7^

**Table 4:** The basic properties of SiO_2_-P25 hybrid nanofluids for different concentrations.

Temperature °C	Ρ g/cm^3^	k W/mK	µ [mPas]	c_p_ J/gK
0.50 vol% SiO_2_-P25				
20	1.033	0.510	1.789	3848.04
30	1.030	0.535	1.470	3863.36
40	1.026	0.549	1.152	3887.89
50	1.022	0.561	0.978	3903.14
60	1.017	0.568	0.815	3925.54
1.00 vol% SiO_2_-P25				
20	1.044	0.522	2.054	3799.25
30	1.041	0.546	1.594	3814.19
40	1.037	0.56	1.313	3838.14
50	1.033	0.571	1.079	3852.96
60	1.028	0.578	0.885	3874.74
1.50 vol% SiO_2_-P25				
20	1.055	0.531	2.281	3733.92
30	1.052	0.553	1.825	3766.05
40	1.048	0.568	1.445	3789.45
50	1.045	0.579	1.212	3803.85
60	1.039	0.587	1.008	3825.04

**Table 5:** The *Pr* numbers of SiO_2_-P25 hybrid nanofluids for different concentrations.

		Concentrations		
		0.50%	1%	1.50%
		Pr Numbers		
Temperature, °C	20	13.50	14.95	16.04
	30	10.62	11.14	12.43
	40	8.16	9.00	9.64
	50	6.80	7.28	7.96
	60	5.63	5.93	6.57
	70	5.05	5.57	6.00
	80	4.37	4.83	5.20
	90	3.86	4.26	4.59
	100	3.45	3.80	4.10

Gray background shows the predicted values.

**Table 6:** The entry length variation D = 0.016 m.

Re	*L_e_/D*, [27]	*L_e_, m* [29]	*L_e_/D,* [28]
5000	18.2	0.29	11.4
8000	19.7	0.31	12.9
11,000	20.8	0.33	13.9
14,000	21.6	0.35	14.8
17,000	22.3	0.36	15.5

**Table 7:** Nu numbers for constant and variable properties of constant HF.

	0.5%		1.0%		1.5%	
Re	Const.	Variable	Const.	Variable	Const.	Variable
5000	43.4	43.7	44.1	44.5	45.6	45.5
8000	63.1	64.2	64.2	65.3	66.4	66.9
11,000	81.9	83.3	83.4	84.8	86.4	86.9
14,000	100.0	101.5	101.8	103.4	105.5	105.9
17,000	117.5	119.0	119.7	121.2	124.1	124.2

**Table 8:** Nu numbers for constant and variable properties for constant wall temperature.

	0.5%		1.0%		1.5%	
Re	Const.	Variable	Const.	Variable	Const.	Variable
5000	47.2	47.2	48.2	48.6	50.2	50.0
8000	69.2	70.1	70.7	72.6	73.8	74.8
11,000	90.7	91.6	92.7	95.2	96.9	98.3
14,000	111.6	112.1	114.1	117.0	119.3	120.8
17,000	131.8	132.0	134.8	138.1	141.0	142.7

**Table 9:** The friction factors by different correlations and the CFD simulations.

Case Identification		No.	Friction Factors	
			Re = 5000	Re = 17,000
Numbers of Table 3 correlations	1	1	0.037626513	0.027709216
	2	2	0.037458997	0.027222256
	3	3	0.038356659	0.027742485
	4	4	0.037458997	0.027222256
	5	5	0.037777816	0.027226969
	6	6	0.037893426	0.026929325
	7	7	0.038619473	0.027272146
	8	8	0.037320168	0.026953634
Constant HF non-variable properties	0%	9	0.036984055	0.027878525
	0.5%	10	0.036983276	0.027878481
	1%	11	0.036983498	0.02787853
	1.5%	12	0.036983328	0.027878432
Constant wall temperature non-variable properties	0%	13	0.036984055	0.027878525
	0.5%	14	0.036983276	0.027878481
	1%	15	0.036983498	0.02787853
	1.5%	16	0.036983328	0.027878432
Constant HF variable properties	0%	17	0.037219022	0.028036842
	0.5%	18	0.03727405	0.028011376
	1%	19	0.037350228	0.028012885
	1.5%	20	0.036843771	0.02796081
Constant wall temperature variable properties	0%	21	0.036975466	0.027986751
	0.5%	22	0.036819132	0.027951593
	1%	23	0.036852728	0.027979754
	1.5%	24	0.036324745	0.027667343

## Nomenclature

HN	Hybrid nanofluid
HT	Heat transfer
HF	Heat flux
CFD	Computer fluid dynamics
EG	Ethylene glycol
PD	Pressure drop
MWCNT	Multi wal carbon nanotubes
Re	Reynolds number
Nu	Nusselt number
Pr	Prandtl number
